# Effect of a mHealth intervention on health literacy in patients completing cardiac rehabilitation: A randomized controlled trial with one- and five-year follow-up

**DOI:** 10.1016/j.ijcrp.2025.200445

**Published:** 2025-06-06

**Authors:** Pernille Lunde, Hanne Søberg Finbråten, Are Hugo Pripp, Birgitta Blakstad Nilsson, Jostein Grimsmo, Asta Bye

**Affiliations:** a Department of Rehabilitation Science and Health Technology, Oslo Metropolitan University, PB 4, St.Olavs plass, Oslo, 0130, Norway; b Department of Health and Nursing Sciences, University of Inland Norway, PB 400, Elverum, N-2418, Norway; c Oslo Centre of Biostatistics and Epidemiology, Oslo University Hospital, PB 4950 Nydalen, Oslo, 0424, Norway; d Division of Medicine, Department of Clinical Services, Oslo University Hospital, PB 4950 Nydalen, Oslo, 0424, Norway; e Department of Cardiac and pulmonary Rehabilitation, Lovisenberg Rehabilitation, Cathinka Guldberg's Hospital, Ragnar Strøms Veg 10, Jessheim, 2067, Norway; f Department of Nursing and Health Promotion, Oslo Metropolitan University, PB 4 St.Olavs plass, Oslo, 0130, Norway; g European Palliative Care Research Centre (PRC), Department of Oncology, Oslo University Hospital and University of Oslo, PB 4950, Nydalen, Oslo, 0424, Norway

**Keywords:** Health literacy, Cardiac rehabilitation, Secondary prevention, mHealth, Randomized controlled trial

## Abstract

**Background and aims:**

Adherence to treatment is a significant challenge for patients with cardiac disease. Optimizing health literacy (HL) is essential in this context. Mobile health (mHealth) interventions have been suggested to improve both treatment adherence and HL. This study aimed to examine the effect of a one-year mHealth intervention on HL and to compare HL changes between the intervention- and the control group.

**Methods:**

This randomized controlled trial included patients completing cardiac rehabilitation, who were randomly allocated to either an intervention group receiving individualized follow-up via an app for one year or a control group receiving usual care. From one-year follow-up to the five-year follow-up, both groups received usual care. HL was measured using the HLS-Q12. Mixed model for repeated measurements and Wilcoxon signed rank test were used to analyse differences between groups, while paired sample *t*-test and Kendall's Tau b correlation analysed within-group changes.

**Results:**

A total of 113 patients were included in the study. No statistically significant differences between the groups were found in total HLS-Q12 score or at item level at any follow-up. However, a statistically significant within-group improvement was observed in the intervention group for the total score (mean change of 2.5 ± 4.6, p < 0.01) and several HLS-Q12 items from baseline to one-year follow-up.

**Conclusions:**

The one-year mHealth intervention did not show an effect on HL levels at one- or five-year follow-ups. However, significant within-group HL improvement from baseline to one-year follow-up suggests that mHealth interventions may have the potential to enhance HL.

## 1 Introduction

Adherence to treatment, both pharmacological and non-pharmacological such as maintaining healthy diet, physical activity, exercise and smoking cessation, is the most important modifiable factor that enhances treatment outcomes in patients with cardiovascular disease (CVD) [1,2]. Adherence has been defined by the World Health Organization as *“the extent to which a person′s behaviour – taking medication, following a diet and/or executing changes, corresponds with agreed recommendations from a health care provider”* [2, p. 3]. In contrast to compliance, adherence implies active participation in treatment collaboration and persistence in practicing and maintaining desired behaviour [3].

In 2023, the European Society of Cardiology (ESC) published a consensus document that reappraises the concept of adherence and offers simple, practical, and feasible suggestions to achieve optimal adherence in the clinical setting, focusing on evidence-based concepts [1]. To increase adherence in CVD, actions should be directed towards five dimensions: the patient, the healthcare provider, the therapy, the disease and the healthcare system [1, p. 152]. The patient dimension includes health literacy (HL), self-efficacy and empowerment.

Sørensen et al. [4] have defined HL as the comprehension and utilization of health information by individuals. It encompasses their knowledge, motivation and competencies to access, understand, appraise and apply health information. This enables them to make informed judgements and decisions in everyday life regarding health care, disease prevention and health promotion to maintain or improve their quality of life throughout their lifespan [4, p. 3].

In patients with CVD, low levels of HL found to be associated with limited disease understanding, suboptimal blood pressure regulation, and inadequate self-management behaviours i.e. exercise routines and bodyweight monitoring, as well as decreased quality of life [5,6]. Additionally, individuals with low HL have been found to be at greater risk of readmission and have higher mortality rates [7]. Studies also suggest an association between HL and enhanced self-efficacy, i.e. belief in the ability to successfully perform and complete tasks or achieve goals [1,8]. Despite being positive towards preventive lifestyle changes, these individuals often struggle to implement them [9].

Over the past decade, mHealth interventions that use mobile technology to provide accessible health information, deliver support, and enhance patient engagement, have been suggested to improve treatment adherence and HL [1,10]. In 2020, we conducted a randomized controlled trial (RCT) to evaluate the effect of a one-year mHealth intervention on adherence to healthy behaviours post-CR. The study showed statistically significant differences favouring the intervention group (IG) over the control group (CG), in terms of exercise capacity, -performance, -habits, and perceived goal achievement after one year [11]. However, a recent five-years follow-up revealed that most of the benefits had diminished [12]. Prior to the RCT, we measured HL before and after CR in a prospective cohort study and found that HL had improved during CR [13]. It remains to be examined what impact the one-year mHealth intervention had on HL, and whether possible effects were sustained at five-year follow-up. Therefore, the primary aim of the present study was to examine the effect of the one-year mHealth intervention on HL compared to usual care, at the end of the intervention and five-years after inclusion to the RCT (five-years follow-up). Additionally, to better understand the potential benefits of mHealth interventions on HL, we aimed to compare and evaluate HL changes in the IG and the CG.

## 2 Methods

All patients in the study were included in a RCT immediately after completing CR. The details of design, methods, sample size, randomization and organization have been previously published [14], as well as one- and five-years results on other outcomes than HL [11,12].

### 2.1 Design

Patients were randomly allocated to one of the two groups via concealed allocation right after completion of CR (baseline assessment). A computer-generated, permuted block randomization scheme, stratified for CR program, was used with a 1:1 allocation ratio. The IG received individualized follow-up via an app for one year after completing the CR program, while the CG received usual care. From the end of the intervention after one-year (one-year follow-up), both groups received usual care. They were approached again for the five-years follow-up by the first author (PL) through e-mail and SMS.

The Regional Committee for Medical and Health Research Ethics (South-East ID: 2016-1476) approved the study protocol. The study was conducted in accordance with the Helsinki Declaration. All patients provided informed, written consent before inclusion to the study. The study protocol which initially included the one-year follow-up assessment only, was registered in ClinicalTrials.gov (NCT03174106). The five-year follow-up trial was registered separately in ClinicalTrials.gov (NCT05697120).

### 2.2 Setting and participants

Patients were recruited from two CR-centers in the eastern part of Norway, whereof one of the centers offered one- and four-week residential CR and the other center offered 12-weeks outpatient CR. All CR-programs were funded by the regional health authority. Inclusion criteria was patients completing CR, age of 40 or above, owner and user of an Android or Apple smartphone; and able to read and understand Norwegian or English. Exclusion criteria were decided primarily based on peak oxygen uptake being the primary outcome of the study and included ischemia or arrythmias uncovered at cardiopulmonary exercise test that gave restrictions equivalent to <80 % of maximal heart rate or Borg Scale (6–20) [15] <15 at exercise. In addition, patients with muscular or skeletal disorders that affected exercise capacity more than the heart disease were excluded, as well as patients with severe malignant diseases, i.e. advanced cancer, that affected the patient's lifespan to a greater extent than their cardiac disease.

Sample size was calculated from the primary outcome, peak oxygen uptake (VO_2peak_) [14]. With a power of 0.8 and a significance level of 0.05, 47 patients in each group was calculated to be sufficient. To allow for a 20 % dropout at one-year follow-up, we aimed to include 113 patients in total. Sample size calculations has not been made for secondary outcomes.

### 2.3 Intervention

Following the baseline assessment, patients in the IG were given access to an app and trained to use it. The app allowed users to set and monitor goals with tasks and accompanying automated reminders. The app was configured with each patient's goals and tasks for the subsequent year. Patients could set the frequency and timing of reminders of the tasks, make notes, and ask questions to the supervisor.

The supervisor, who conducted the baseline assessment and taught patients in how to use the app, had access to an administrator interface for monitoring patient progress and sending brief motivational messages directly in to the patients' app.

Weekly individualized feedback was provided for the first 12 weeks, followed by monthly feedback for rest of the year. All feedback was based on patient goals and notes as well as completed and pending tasks. Additionally, patients received one to three tailored motivational messages every week throughout the year. Any questions from the patients were responded to within two working days. The individualized follow-up continued until the one-year follow-up assessment.

At one-year follow-up, the app was removed from the patients' smartphones and the follow-up discontinued. Patients were then encouraged to maintain or improve their health based on their individual risk profile, and to visit their general practitioner (GP) and cardiologist when needed. A detailed description of the intervention has previously been published [14].

### 2.4 Control group

Patient allocated to the CG received usual care which included visiting their GP and cardiologist if, and when needed. After baseline assessment and at one-year follow-up they were encouraged to maintain or improve their healthy behaviour based on their own goals and individual risk profile.

### 2.5 Assessments

Assessments were performed at baseline, after one year and after five years at the same CR centre where the patient was recruited. During baseline assessment, demographic data were collected. In addition, all assessment included a cardiopulmonary exercise test, including blood pressure measurements and bodyweight, conversation with a researcher collecting data such as exercise habits, cholesterol levels and so on, and completion of standardized questionnaires. All assessments were conducted by the same researcher (PL) in all included patients. However, this researcher was not present during the cardiopulmonary exercise test. This to keep the assessors of the primary outcome of the study blinded for group allocation. Results regarding primary outcome and most of the secondary outcomes have previously been reported [11,12], except from HL.

#### 2.5.1 Health literacy

A short version of the European Health Literacy Survey Questionnaire [16,17], the HLS-Q12 [18] was used to measure HL. This questionnaire reflects the conceptual model of HL developed by Sørensen et al. [4] and measures HL proficiency across four cognitive domains (access, understand, appraise and apply health information) and three health domains (health care, disease prevention and health promotion). The HLS-Q12 consists of 12 items and offers four response categories from 1) very difficult to 4) very easy. A total score ranging from 12 to 48 is provided, where higher score indicates higher HL proficiencies. The HLS-Q12 has previously been validated in people with type 2 diabetes [19], in the general Norwegian population [18], and recently, in people participating in CR [13].

### 2.6 Statistical analyses

IBM SPSS Statistics (version 29) and STATA (version 18) were used for statistical analysis. Continuous, normally distributed baseline data were analysed with an independent *t*-test to test for differences between groups, and Pearson's chi-squared test was used to analyse the categorical data. Strategies for dealing with missing data in clinical trials [20] was applied. Differences between groups in the total score of HLS-Q12 were assessed using a mixed model for repeated measurements with a subject-specific random intercept and group, follow-up time, interaction between group and follow-up time, and HLS-Q12 total score at baseline as fixed effects. Differences between groups in items at each follow-up time were analysed using Wilcoxon signed rank test due to ordinal data. Paired sample *t*-test was applied to analyse within group changes for the total score of HLS-Q12. Kendall's Tau b correlation analysis was applied to analyse within group changes for each item. Analysis was carried out by intention-to-treat and all tests were two-sided. Data are presented as mean ± standard deviation (SD) unless stated otherwise. A p-value <0.05 was considered statistically significant.

## 3 Results

**Fig. 1:**
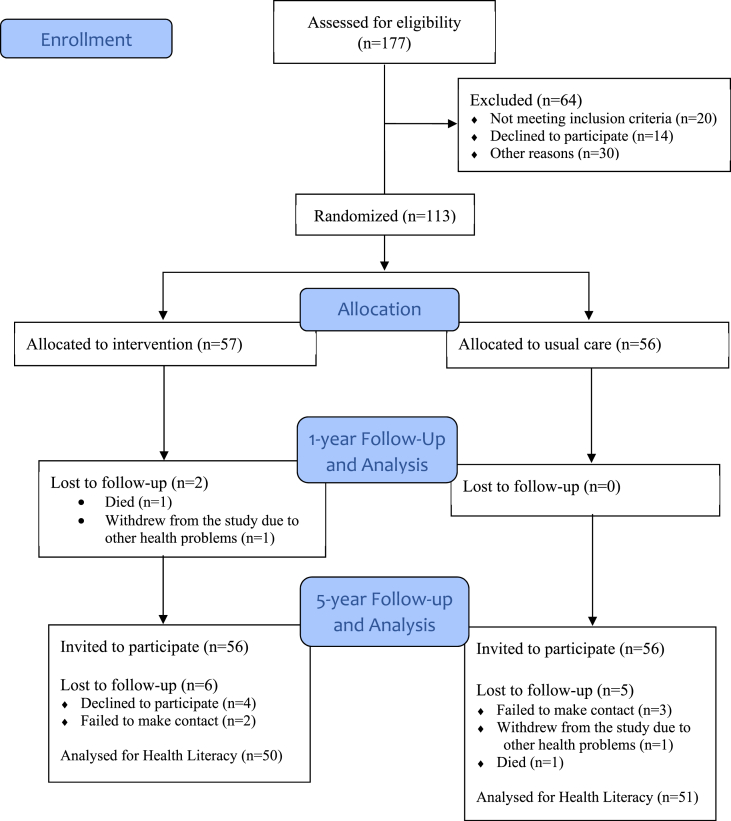
The CONSORT flow diagram.

In total, 113 of 177 patients screened for eligibility were randomized to the IG or the CG (Fig. 1). One- and five-year follow-up was completed in June 2019 and September 2023 respectively. In total, 113 patients responded to the HLS-Q12 at baseline, 111 at one-year and 101 at five-year follow-up.

Descriptive statistics of the sample are presented in Table 1. There were no statistically significant differences in baseline characteristics between the IG and CG.

**Table 1:** Baseline characteristics of the sample.

	Total (n = 113)	Control group (n = 56)	Intervention group (n = 57)
Age	59.0 ± 8.7	58.4 ± 8.2	59.5 ± 9.1
Female, n (%)	25 (22.1)	16 (28.6)	9 (15.8)
Married or cohabitant, n (%)	89 (78.8)	43 (76.8)	46 (80.7)
Current smoker, n (%)	4 (3.5)	1 (1.8)	3 (5.3)
Bodyweight	90.2 ± 16.9	88.5 ± 17.0	91.8 ± 16.8
Education	2.9 ± 2.7	3.0 ± 2.8	2.9 ± 2.6
Type D personality, n (%)	13 (11.5)	8 (14.3)	5 (8.8)
Disease, n (%)			
ACS	44 (38.9)	22 (39.3)	22 (38.6)
CAD	39 (34.5)	19 (33.9)	20 (35.1)
Valve	19 (16.8)	8 (14.3)	11 (19.3)
Other	11 (9.8)	7 (12.5)	4 (7)
Treatment, n (%)			
PCI	55 (48.7)	26 (46.4)	29 (50.9)
CABG	22 (19.5)	12 (21.4)	10 (17.5)
Valve surgery	19 (16.8)	8 (14.3)	11 (19.3)
Conservatively	10 (8.8)	6 (10.7)	4 (7.0)
Other	7 (6.3)	4 (7.2)	3 (5.3)
Medication, n (%)			
Betablocker	69 (61.1)	32 (57.1)	37 (64.9)
Statins	96 (85)	45 (80.4)	51 (89.5)
ASA + Plate inhibitor	75 (66.4)	39 (69.6)	36 (63.2)
Antihypertensive	55 (48.7)	29 (51.8)	26 (45.6)
Type of CR, n (%)			
One week	35 (31)	17 (30.4)	18 (31.6)
Four weeks	40 (35.4)	20 (35.7)	20 (35.1)
Twelve weeks	38 (33.6)	19 (33.9)	19 (33.3)
CPET			
VO_2peak_ (mL·kg^−1^·min^−1^)	29.6 ± 7.7	29.9 ± 6.7	29.4 ± 8.7
VO_2peak_ (L·min^−1^)	2.64 ± 0.74	2.63 ± 0.75	2.65 ± 0.74
Total score of HLS-Q12	37.5 ± 4.7	38.1 ± 4.5	36.9 ± 4.8

Values are mean ± standard deviation or n (%), unless otherwise stated. Education: years beyond upper secondary school; ACS: acute coronary syndrome; CAD: coronary artery disease; PCI: percutaneous coronary intervention; CABG: coronary artery bypass graft; ASA: acetylsalicylic acid, CR: cardiac rehabilitation; CPET: cardiopulmonary exercise test; VO_2peak_: peak oxygen uptake; HLS-Q12: a 12-items short version of the European Health Literacy Survey Questionnaire.

The mixed model for repeated measurements analyses did not show any statistically significant differences between the groups in the total score of HLS-Q12 at one- or five-year follow-up (mean difference at one-year follow-up: 1.04, 95 % confidence interval (CI) −0.56 – 2.65, *p* = 0.202, mean difference at five-year follow-up: 0.41, 95 % CI -1.26 – 2.07, *p* = 0.630). The Kendall's tau b correlation analysis did not show any statistically significant differences between the groups in any of the items of the HLS-Q12 at any of the follow-up times.

**Fig. 2:**
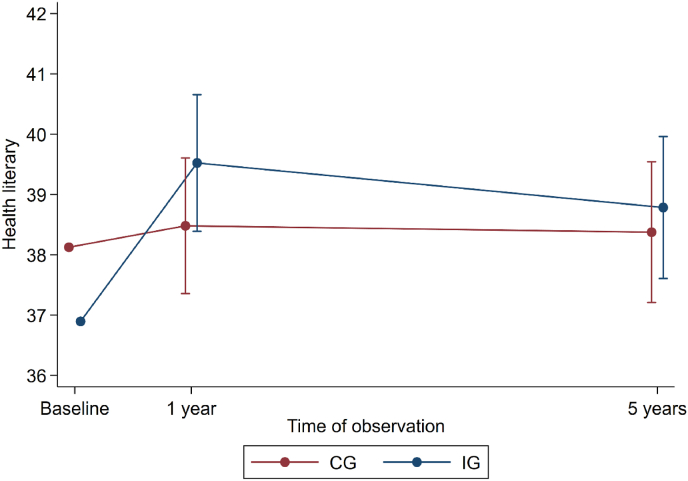
Health Literacy mean scores in the intervention and control groups at baseline, one- and five-year follow-up.

At baseline, the total score of HLS-Q12 was lower in the IG than in the CG (36.9 ± 4.8 in the IG vs 38.1 ± 4.5 in the CG) (Fig. 2). However, this difference was not statistically significant (*p* = 0.162). From baseline to the one-year follow-up, the IG had a statistically significant within group improvement in the total score of HLS-Q12 (from 36.9 ± 4.8 to 39.2 ± 4.9, mean change of 2.5 ± 4.6, *p* < 0.01), while the CG remained stable (from 38.1 ± 4.5 to 38.9 ± 5.0). From one-year follow-up to five-year follow-up, the total score of HLS-Q12 slightly decreased for the IG (from 39.2 ± 4.9 to 38.2 ± 5.0), whereas the score maintained for the CG (from 38.9 ± 5.0 to 38.8 ± 5.3).

The questions in the HLS-Q12 as well as the proportion of patients that answered difficult or very difficult are presented in Table 2. In addition to the statistically significant improvement of the total HLS-Q12 score within the IG from baseline to one-year follow up, items no 1, 2, 5, 8, 9 and 11 also improved statistically significantly. The score of items 1, 9 and 11 also improved statistically significant within the CG. From one-year follow-up to the five-year follow-up, the score of item 7 statistically significantly (*p* = 0.037) improved within the IG, while the score of item 12 was statistically significantly worsened (*p* = 0.019). From baseline to five-year follow-up, item 2 (*p* = 0.012), item 7 (*p* < 0.001), as well as the total score (*p* = 0.02) of the HLS-Q12 improved within the IG. In the CG, the score of item 1 (*p* = 0.005) and 9 (*p* = 0.018) statistically significantly improved from baseline to the five-year follow-up.

**Table 2:** The proportion of patients (%) in each group that answered (very) difficult to the HLS-Q12 items.

		Item wording	Baseline (n = 113)		One-year follow-up (n = 111)		Five-year follow-up (n = 101)	
Item no	HLS_19_/HLS-EU item no	How easy would you say it is	Control group (n = 56)	Intervention group (n = 57)	Control group (n = 56)	Intervention group (n = 55)	Control group (n = 51)	Intervention group (n = 50)
**1**	CORE-HL2	… to find information on treatments of illnesses that concern you?	11	14	0	4	2	12
**2**	CORE-HL7	… to understand what to do in a medical emergency?	11	14	11	4	16	12
**3**	CORE-HL10	… to judge the advantages and disadvantages of different treatment options?	14	30	23	22	26	22
**4**	CORE-HL14	… to follow instructions on medication?	2	7	5	7	10	14
**5**	CORE-HL18	… to find information on how to handle mental health problems like stress or depression?	16	19	14	13	18	24
**6**	CORE-HL23	… to understand why you need health screenings?	0	5	5	2	2	6
**7**	CORE-HL28	… to judge if the information on health risks in the mass media is reliable?	21	37	20	31	28	18
**8**	CORE-HL30	… to decide how you can protect yourself from illness based on advice from family and friends?	21	28	16	22	20	24
**9**	CORE-HL32	… to find information on healthy life styles such as physical exercise, healthy food and nutrition?	2	2	2	4	0	4
**10**	CORE-HL38	… to understand information on food packaging?	21	26	21	22	24	20
**11**	CORE-HL43	… to judge which everyday behaviour is related to your health?	5	11	5	4	6	6
**12**	CORE-HL44	… to make decisions to improve your health?	12	14	11	9	12	18

## 4 Discussion

To our knowledge, the present study is the first study evaluating the effect of a mHealth intervention on HL in patients with CVD, specifically post-CR. Our main finding was non-significant difference between the groups at one- and five-years follow-up. However, a statistically significant improvement in HL was observed within the IG from baseline to one-year follow-up. In contrast, the level of HL in the CG remained stable from baseline through both one- and five-year follow-up.

There may be numerous reasons why the intervention did not lead to statistically significant differences in HL between the IG and the CG. As previously described [11], the mHealth intervention evaluated in the present study can be considered as a complex intervention. Several factors determine whether an intervention is complex or not, for example the number of components involved in the intervention; the range of behaviours targeted; expertise and skills needed by those delivering and receiving the intervention; number of groups, settings or levels targeted; or the permitted level of flexibility of the intervention or its components [21]. The mHealth intervention evaluated in the present study was primarily based on providing individualized feedback on patients own goals and their specific needs [14] and considered each patient stage of change according to the transtheoretical model of change [22]. Although the feedback provided by the supervisor considered the patient's level of HL, the mHealth intervention was aimed at promoting adherence to healthy behaviours initiated or adapted through CR, and not to improve level of HL. This is important as an intervention should be targeted the specific behaviour of interest to verify the real potential and effect of a complex intervention [21].

Another potential cause for the lack of effect includes the relatively small sample in the present study. The power calculation was conducted based on the primary outcome, VO_2peak_, as this was considered as the most appropriate outcome to measure adherence to healthy behaviour post-CR [14]. Therefore, we cannot rule out that the null hypothesis has been kept despite a real difference between the groups. To investigate whether the lack of effect could be due to a statistical type II error, the minimal important difference (MID) must be known. Currently, this has not been investigated for HLS-Q12. As a starting point for estimating the MID of quality of life (QoL) scales, researchers have been encouraged to use 0.5 SD in patients with chronic diseases [23]. As both QoL and HL are multidimensional constructs, it can be argued that using 0.5 SD as MID can be applied for HL as well. At baseline, mean total score of HLS-Q12 was 37.5 ± 4.7 for the whole sample. By assuming that 0.5 SD can be used for estimating MID in HLS-Q12, we can argue that the mHealth intervention used in the present study had a clinically significant impact on HL since we found a statistically significant improvement in the total score of HLS-Q12 for the IG from baseline to one-year follow-up with a mean change of 2.5 ± 4.6, (*p* < 0.01). Considering the mHealth interventions' aim in the present study, we will argue that this finding points in the direction that mHealth interventions can have a great potential to support and improve patients in terms of improving their HL. This is supported by Lin and Lou [24] which conclude in their systematic review, examining the effect of mHealth-based interventions on HL and related factors, that mHealth delivered via mobile phone applications is effective in improving patient HL. However, it should be interpreted with caution as the evaluation of mHealth interventions regarding HL appears to be in its early days, in particular for patients with non-communicable diseases, such as CVD. For these populations, we believe it is important to consider the patients HL at baseline, and to tailor the mHealth intervention individually. In our sample, the person behind the app was highlighted as crucial for success regarding adherence to healthy behaviour [25]. We believe that this also is of great importance in mHealth interventions aiming to improve patients' HL because individualized feedback, based on patients initial level of HL, increases the likelihood that the information and feedback will be understandable and perceived as meaningful and motivating rather than annoying [25].

Despite that most benefits gained in CR have previously been described to dimmish >6 months post-CR [26,27], it is gratifying that improvements in HL gained through CR [13] seems to maintain or improve in our sample. This may indicate that the level of HL is something that is acquired to a greater extent compared to lifestyle habits such as physical activity, exercise and healthy nutrition. However, despite an improvement in HL that were maintained at 5 years follow-up, these patients demonstrated poor adherence to both pharmacological and non-pharmacological treatment which was expressed, among other things, by not achieving the treatment goal for LDL-cholesterol and a decline in VO_2peak_, respectively [12]. Therefore, considering that the proportion of patients that answered (very) difficult (Table 2) was lower in most questions of the HLS-Q12 compared to national normative data (n = 2999) [28] at both baseline, one- and five-year follow-up, it can be questioned to what degree level of HL associates with adherence to treatment.

### 4.1 Strengths and limitations

To the best of our knowledge, this is the first study evaluating HL in a post-CR population and evaluating the effect of a mHealth intervention proven to be effective in terms of adherence to healthy behaviour [11]. A strength of our study was the broad inclusion criteria, which allowed the majority of patients participating in CR to be included in the study. This, along with the inclusion of patients from various CR programs, is significant as it increases the generalizability of our results to the broader population of patients completing CR. However, findings from the present study must be interpreted with caution since HL was a secondary outcome. Consequently, the relatively small sample size means we lack sufficient statistical power which further implies that the risk of statistical errors, particularly a type II error, is high. Nevertheless, the results highlight that mHealth interventions are likely to have significant impact in improving patients' HL, and our findings could be highly relevant in the planning of future studies in this field. Lastly, measuring and evaluating multidimensional concepts like HL is challenging. By using the carefully developed and evaluated questionnaire HLS-Q12, we will claim that measuring HL has been done in best possible way.

### 4.2 Conclusion

One-year follow-up with an mHealth intervention did not reveal any effect on level of HL, either at one- or five-year follow-up. However, a statistically significant improvement of HL was found within the IG from baseline to one-year follow-up (end of mHealth intervention). This may indicate that mHealth interventions have the potential to positively influence the level of HL. Considering that mHealth interventions are complex interventions, and that our mHealth intervention was not specifically developed to improve HL, future research should strive to develop and evaluate the effect of a tailored mHealth intervention in terms of potential influence on level of HL. This to reveal the real potential of such interventions on the level of HL. Finally, to understand the true significance of HL in terms of treatment adherence, studies with sufficient statistical power are needed.

## CRediT authorship contribution statement

**Pernille Lunde:** Writing – review & editing, Writing – original draft, Project administration, Methodology, Investigation, Funding acquisition, Formal analysis, Data curation, Conceptualization. **Hanne Søberg Finbråten:** Writing – review & editing, Visualization, Validation, Methodology, Conceptualization. **Are Hugo Pripp:** Methodology, Formal analysis. **Birgitta Blakstad Nilsson:** Writing – review & editing, Methodology, Conceptualization. **Jostein Grimsmo:** Writing – review & editing, Investigation. **Asta Bye:** Writing – review & editing, Methodology, Conceptualization.

## Declaration of competing interest

The authors report no relationships that could be construed as a conflict of interest.
